# Single-Shot Sub-Picosecond Ultrafast Microscopic Imaging Utilizing Spatial-Frequency Multiplexing for Ultrafast Laser-Induced Plasma Visualization

**DOI:** 10.3390/nano15181410

**Published:** 2025-09-12

**Authors:** Hang Li, Yahui Li, Yang Shang, Mengmeng Yue, Duan Luo, Yanhua Xue, Guilong Gao, Jinshou Tian

**Affiliations:** 1 State Key Laboratory of Ultrafast Optical Science and Technology, Xi’an Institute of Optics and Precision Mechanics, Xi’an 710119, China; lihang@opt.ac.cn (H.L.); luoduan@opt.ac.cn (D.L.);; 2 Collaborative Innovation Center of Extreme Optics, Shanxi University, Taiyuan 030006, China

**Keywords:** single-shot ultrafast imaging, spatial-frequency multiplexing, spatial resolution, ultrafast laser-induced plasma

## Abstract

Ultrafast laser processing can produce micro/nanostructures, which is of great interest in advanced manufacturing. Ultrafast laser-induced events include non-equilibrium dynamic phenomena, occurring on the femtosecond to picosecond time scale and nanometer to micron space scale. Single-shot ultrafast imaging can provide multiple time-correlated evolution frames in one non-repeatable event with a temporal resolution of sub-picoseconds. However, previous approaches suffer from degraded spatial resolution, which is a bottleneck in microscopic imaging. For the spatial-frequency multiplexing methods based on structured illumination, a reconstruction strategy was proposed utilizing the frames’ conjugate symmetry in the Fourier domain. The spatial resolution is double that of the traditional algorithm by evaluating with synthetic data, revealing that the reconstruction resolution can reach the diffraction limitation. A two-frame microscopic system was constructed with a frame interval of 300 fs and a maximum spatial resolution of 1.4 μm. The interaction between a femtosecond laser and a fused silica glass plate was captured in a single shot and the dynamic evolution of the induced plasma was observed, verifying the application feasibility in ultrafast laser processing, providing experimental observations for interaction mechanism research and theoretical model optimization.

## 1. Introduction

Ultrafast laser processing holds great promise in high-end manufacturing due to its micrometer- and even nanometer-scale precision, benefiting fields such as semiconductors, microelectronics, medical devices, and micro/nanophotonics [1,2,3,4,5]. The dynamic, multi-scale phenomena triggered by ultrafast laser–matter interactions critically influence processing outcomes. These interactions begin with multi-photon and avalanche ionization, along with inverse Bremsstrahlung absorption, generating highly non-equilibrium electronic states. Subsequent energy transfer to the lattice leads to melting, vaporization, plasma formation, and re-solidification [6,7]. The intrinsic properties of materials (e.g., metals, semiconductors, ceramics, polymers, and biological tissues) and laser parameters (pulse energy, duration, repetition rate, wavelength, etc.) significantly affect the underlying mechanisms, enabling precise control over microstructures and functions [7,8]. For instance, ultrafast laser irradiation of transparent glass yields different outcomes [9,10,11], refractive index changes, nanogratings, or nanovoids depending on the pulse energy density. These mechanisms underpin advanced fabrication techniques such as direct waveguide writing, diffractive element fabrication, and 3D optical data storage. In metals [12,13,14,15], ultrafast laser ablation is dominated by spallation and phase explosion, supporting functional surface microstructuring for applications like anti-icing and drag reduction.

Despite these advances, the fundamental mechanisms during the earliest stage of interaction, especially the nonlinear absorption of laser energy and the highly non-equilibrium electronic states within the first few picoseconds [6], remain elusive due to the lack of systematic theoretical models and experimental tools. This early phase governs subsequent thermo-dynamic evolution (e.g., phase transitions and defect formation) on nanosecond to microsecond scales, and its poor characterization limits precision and predictability in processing. Thus, there is a critical need for diagnostic techniques with sub-picosecond temporal and nano/sub-micron spatial resolution to visualize these transient non-equilibrium processes and to refine theoretical models and process parameters.

Pump–probe techniques, widely used to investigate ultrafast phenomena with femtosecond or even attosecond temporal resolution, have been instrumental in this area [16,17]. A pump laser initiates the dynamic process, while a time-delayed probe captures temporal snapshots via configurations such as shadowgraphy [18,19], reflectometry [12], interferometry [20], and hybrid modes [21,22]. However, pump–probe approaches require event repeatability, which is often compromised in ultrafast laser–matter interactions due to nonlinear effects, material inhomogeneity, and laser fluctuations. These factors degrade temporal correlation and limit model accuracy.

To overcome these limitations, single-shot ultrafast imaging techniques have emerged, capable of capturing non-repeatable dynamics within a single event. Based on computational imaging with modulated illumination, they achieve interframe intervals from hundreds of femtoseconds to a few picoseconds [23,24,25,26,27,28,29,30,31]. Among them, the structured illumination approach (Frequency Recognition Algorithm for Multiple Exposures, FRAME) [31] is promising for achieving high spatiotemporal resolution. It employs a sequence of structured pulses for target illumination and retrieves time-resolved frames with demultiplexing algorithms in the Fourier domain. In previous studies of femtosecond FRAME, the propagation of light through a Kerr-sensitive medium was visualized with four frames and a frame interval of 200 fs. The temporal resolution of FRAME is determined by the sub-pulse width and the spacing between consecutive sub-pulses. The primary challenges are the sub-pulse spatial encoding strategy and reconstruction algorithm in the Fourier domain, both of which have been systematically investigated in our preliminary studies [32,33]. Experimental validations have demonstrated that FRAME can achieve high spatiotemporal resolution and reconstruction fidelity because the target information is directly extracted from the Fourier domain instead of computational unmixing. However, current implementations of FRAME are limited to macroscopic observations of light transport phenomena with a spatial resolution of ∼0.015 lp/μm over a field of view of 7 × 7 mm^2^, which is not suitable for capturing laser–matter interaction processes typically occurring within micron to sub-micron areas. Therefore, microscopic systems with a smaller field of view and higher spatial resolution are desired.

To address the critical need for monitoring ultrafast laser ablation dynamics, this work investigates the spatial resolution limitation of FRAME, proposes a frame extraction strategy with higher recovered performance, and constructs a microscopic FRAME system with high-frequency structured illumination (1.4 μm spatial resolution). We demonstrate its capability in visualizing the laser-induced process in a fused silica glass plate with a frame interval of 300 fs.

## 2. Theories and Methods

**Figure 1:**
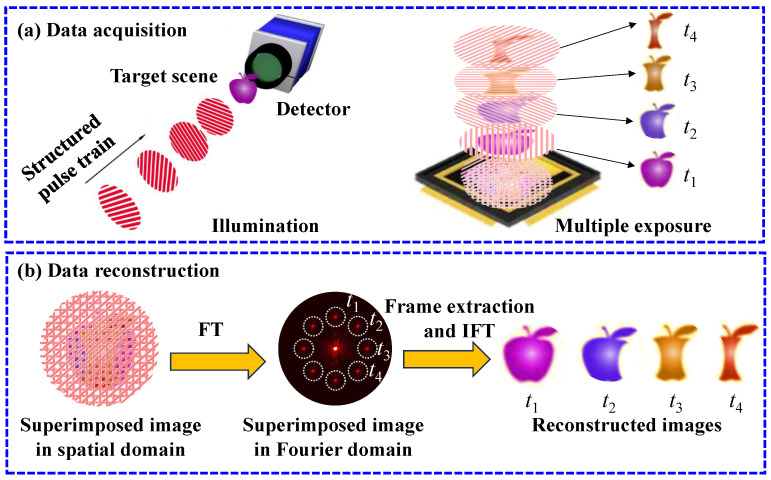
Illustration of the working principle of FRAME. (**a**) Data acquisition. A target scene is illuminated with a structured pulse train. The sub-pulses modulated by the target’s transient states at $t_{1}$–$t_{4}$ are cumulated on a detector, generating a superimposed FRAME image. (**b**) Reconstruction. The superimposed image is transformed into the Fourier domain to extract the target’s temporal information distributed at distinct locations. With the inverse Fourier transformation of the extracted Fourier domain information, the corresponding spatial domain frames at $t_{1}$–$t_{4}$ can be recovered.

The working principle of FRAME is illustrated in Figure 1, including data acquisition and reconstruction stages. For data acquisition (Figure 1a), the target is illuminated with a sequence of structured pulses, where each sub-pulse arrives at the target and is spatially modulated by the target’s transient state at a distinct moment. All modulated sub-pulses are sequentially imaged onto a detector and integrated into a single multiplexed exposure, providing a composite image containing multiple temporally resolved snapshots of the dynamic evolution. The reconstruction algorithm transforms the superimposed image into the Fourier domain and extracts frames around different carrier frequencies, which are uniquely determined by the structured pattern of each pulse. The extraction involves transferring the information around the carrier frequency to the origin, filtering the valid information with a lowpass filter, and performing the inverse Fourier transformation to recover each frame in the spatial domain, as illustrated in Figure 1b.

### 2.1. Illumination Modulation Model

FRAME’s spatial resolution is fundamentally governed by the arranging strategies of carrier frequencies in the Fourier domain. Intuitively, high-fidelity reconstruction can be provided with reduced crosstalk from other frames by increasing the carrier frequencies and the distance between adjacent frames. To assess the feasibility in microscopic applications, the spatial resolution of a FRAME system can be evaluated by considering illumination encoding, coupled imaging, and signal detecting performances.

**Figure 2:**
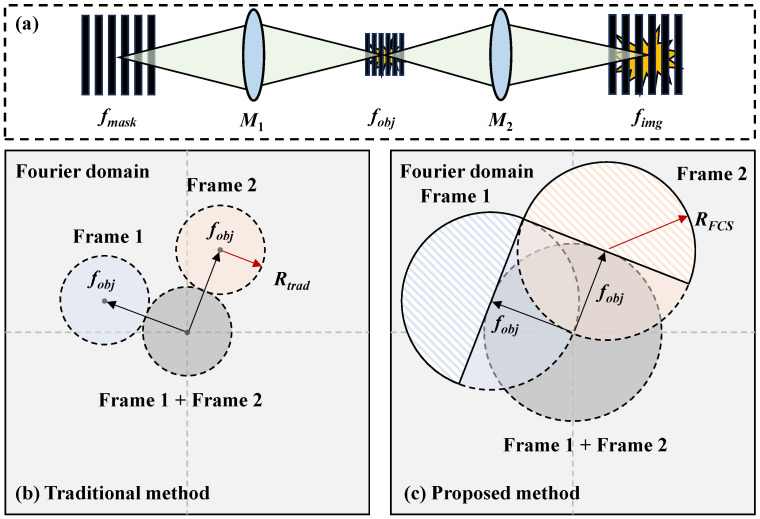
(**a**) Illumination modulation process. A grating pattern with a frequency of $f_{mask}$ is projected onto the target through the first coupling lens with a magnification of $M_{1}$, generating an illumination pattern with a frequency of $f_{obj}$. The illumination pattern is modulated by the object’s transient state and projected onto the detector through the second coupling lens with a magnification of $M_{2}$. The imaged pattern’s frequency on the detector is $f_{img}$. (**b**) Traditional and (**c**) proposed data reconstruction strategies in the Fourier domain on the object plane. The maximum frame extraction filter of the traditional strategy is $R_{trad}=f_{obj}/2$ to avoid crosstalk from the zero-component in gray. The filter can be expanded using the Fourier conjugate symmetry with $R_{FCS}=f_{obj}$. The shaded areas, which are not influenced by the zero-component, are used to improve the spatial resolution.

The illumination modulation process is shown in Figure 2a. Firstly, a grating pattern $P_{mask}\left(x,y\right)$ with a frequency of $f_{mask}$ is projected onto the target through the first coupling lens with a magnification of $M_{1}$, generating an illumination pattern P(x,y) with a frequency of $f_{obj}=f_{mask}/M_{1}$. $P_{mask}\left(x,y\right)$ and P(x,y) can be expressed as

(1) $$P_{mask}\left(x,y\right)=1+Q_{p}\sin\left[2\pi \left(f_{mask}^{x}x+f_{mask}^{y}y\right)\right]$$

(2) $$P\left(x,y\right)=P_{mask}\left(x/M_{1},y/M_{1}\right)$$

where $Q_{p}$ is the modulation degree, θ is the modulation angle, $f_{mask}^{x}=f_{mask}\cos\theta$, and $f_{mask}^{y}=f_{mask}\sin\theta$. Bounded by diffraction limits, the maximum $f_{obj}$ is $f_{DL}$, where $f_{DL}$ is the diffraction limitation 2NA/λ, λ is the illumination wavelength, and NA is the numerical aperture of the projection optics.

Secondly, the pattern *P* is modulated by the object’s transient state $I_{obj}$ and projected onto the detector $I_{img}$ through the second coupling lens with a magnification of $M_{2}$. The imaged pattern’s frequency on the detector is $f_{img}=f_{obj}/M_{2}$. $I_{img}$ can be expressed as

(3) $$I_{img}\left(x,y\right)=P\left(x/M_{2},y/M_{2}\right)\cdot I_{obj}\left(x/M_{2},y/M_{2}\right)$$

To ensure resolvable patterns with a high degree of modulation, the maximum $f_{img}^{max}$ is $1/4d_{px}$, where $d_{px}$ is the detector pixel size, as discussed in [32]. By integrating *N* illumination pulses in a single exposure, the overlapped image $I_{img}^{overlap}\left(x,y\right)$ on the detector can be expressed as

(4) $$I_{img}^{overlap}\left(x,y\right)=\sum_{n}^{N}I_{img}^{n},n=1,\ldots ,N$$

### 2.2. Frame Extraction Algorithms

Taking the Fourier transformation (FT) of $I_{img}^{overlap}\left(x,y\right)$, ${\tilde{I}}_{img}^{overlap}\left(f_{x},f_{y}\right)$ can be written as

(5) $$\begin{array}{cc} {\tilde{I}}_{img}^{overlap}\left(f_{x},f_{y}\right) & =\mathrm{FT}(\sum_{n}^{N}P^{n}\left(x/M_{2},y/M_{2}\right)\cdot I_{obj}^{n}\left(x/M_{2},y/M_{2}\right))\\ & =\sum_{n}^{N}\mathrm{FT}\left(P^{n}\left(x/M_{2},y/M_{2}\right)\right)*\mathrm{FT}\left(I_{obj}^{n}\left(x/M_{2},y/M_{2}\right)\right)\\ & =\sum_{n}^{N}{\tilde{I}}_{obj}^{n}\left(M_{2}f_{x},M_{2}f_{y}\right)*[\delta \left(M_{2}f_{x},M_{2}f_{y}\right)\\ & +\frac{Q_{p}}{2}\left(\delta \left(M_{2}f_{x}-f_{obj}^{n,x},M_{2}f_{y}-f_{obj}^{n,y}\right)+\delta \left(M_{2}f_{x}+f_{obj}^{n,x},M_{2}f_{y}+f_{obj}^{n,y}\right)\right)]\\ & =\sum_{n}^{N}{\tilde{I}}_{obj}^{n}\left(M_{2}f_{x},M_{2}f_{y}\right)\\ & +\frac{Q_{p}}{2}\sum_{n}^{N}{\tilde{I}}_{obj}^{n}\left(M_{2}f_{x}-f_{obj}^{n,x},M_{2}f_{y}-f_{obj}^{n,y}\right)+{\tilde{I}}_{obj}^{n}\left(M_{2}f_{x}+f_{obj}^{n,x},M_{2}f_{y}+f_{obj}^{n,y}\right) \end{array}$$

where the symbol ∗ represents convolution, ${\tilde{I}}_{obj}^{n}$ is the $n^{th}$ object’s frame in the Fourier domain and δ is the Dirac delta function. $f_{obj}^{n,x}$ and $f_{obj}^{n,y}$ are the *x* and *y* components of $f_{obj}^{n}$, respectively. There are three copies of the object’s information at distinct positions in the Fourier domain. The illumination modulation $Q_{p}$ determines the proportions of the zero- and high-carrier-frequency components. A larger $Q_{p}$ ensures less crosstalk from the zero-frequency component, delivering higher reconstruction quality [32]. In Equations (1)–(5), the temporal modulation during the illumination and imaging processes is not included, as the reconstruction from the superimposed FRAME image is insensitive to the temporal properties of the illumination sub-pulses.

For the reconstruction framework, by transferring the components at the high carrier frequency, ($f_{obj}^{n,x}$, $f_{obj}^{n,y}$) or ($-f_{obj}^{n,x}$, $-f_{obj}^{n,y}$), to the origin, ${\tilde{I}}_{obj}^{n}$ can be extracted by adopting a lowpass filter FLT. FLT follows the super-Gaussian distribution,

(6) $$FLT\left(r\right)=\exp\left[-{\left(r/R\right)}^{2\alpha }\right]$$

where *r* is the radius from the origin, *R* is the radius of the filter, and α is the hyperparameter that characterizes the function shape. A larger α leads to sharper rising and falling edges. The suggested α is 2 for optimized reconstruction performance, as investigated in [32].

Figure 2b,c show the principles of traditional and proposed data reconstruction strategies in the Fourier domain on the object plane for a dual-frame system. The gray area represents the zero-component (the first term in Equation (5)), which is the overlap of all frames. The blue and orange areas represent the object’s information at different high carrier frequencies (the second or third term in Equation (5)). The conjugate components are not shown as they contain the same information.

For the traditional method proposed in [31,34,35], as shown in Figure 2b, the frame extraction filter has a maximum radius of $R_{trad}=f_{obj}/2$ to avoid crosstalk from the zero-component in gray. The $n^{th}$ frame is then recovered by applying the inverse Fourier transformation (IFT),

(7) $${\hat{I}}_{obj}^{n,trad}=\mathrm{IFT}\left[{\tilde{I}}_{obj}^{n}\cdot FLT\left(R_{trad}\right)\right]$$

To expand the extraction filter to cover more high-frequency information, we proposed a strategy utilizing the Fourier conjugate symmetry (FCS), which means that half of the information in the filter with negligible crosstalk is enough for high-fidelity reconstruction. The valid extracted areas are the shadowed areas with a maximum filter radius $R_{\mathrm{FCS}}=f_{obj}$, and the complementary information is completed by taking the symmetrically mirroring and conjugating operations, denoted as FCS, as shown in Figure 2c.

(8) $${\hat{I}}_{obj}^{n,FCS}=\mathrm{IFT}\left\{\mathrm{FCS}\left[{\tilde{I}}_{obj}^{n}\cdot FLT\left(R_{\mathrm{FCS}}\right)\right]\right\}$$

Due to $f_{obj}$ being bounded by the diffraction limitation $f_{DL}$, the maximum achievable spatial resolution for a dual-frame system is $f_{DL}/2$ and $f_{DL}$ for the traditional and proposed reconstruction strategies, respectively. With an increase in frame number, spatial resolution decreases because frame extraction filters have to be smaller to suppress the interference of adjacent frames. It should be noted that the object’s information outside the frame extraction filter should be restricted to avoid additional crosstalk by introducing object defocusing blur, while the illumination’s structured patterns are focused though the second coupling lens, as shown in Figure 2a.

## 3. Results

### 3.1. Simulations

**Figure 3:**
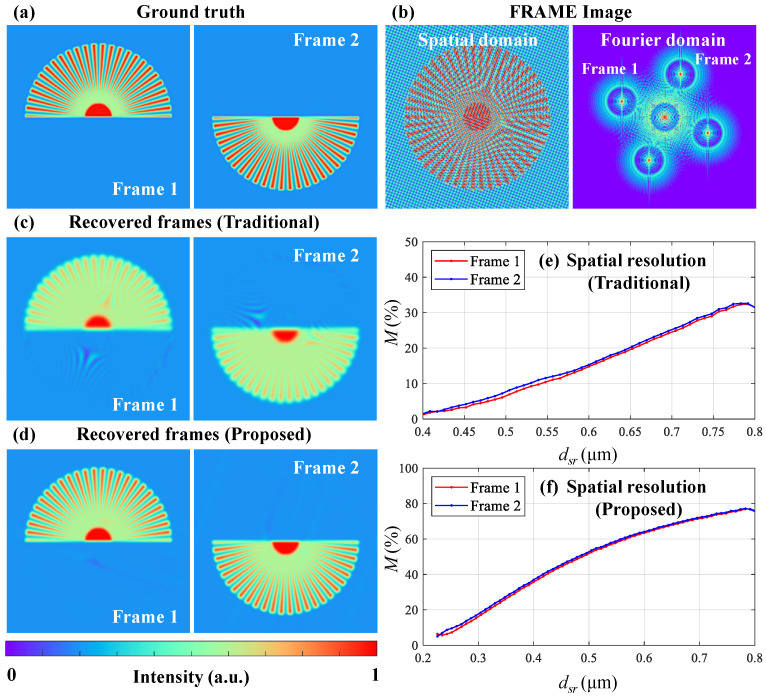
Spatial resolution evaluation using the traditional and proposed FCS reconstruction strategies. (**a**) Ground truth frames. (**b**) Overlapped images from a FRAME system in the spatial and Fourier domains. Recovered frames with the (**c**) traditional and (**d**) proposed algorithms. Relative modulation curves of Frames 1 (red) and 2 (blue) with the (**e**) traditional and (**f**) proposed algorithms.

Figure 3 demonstrates the microscopic imaging performance using the traditional and proposed reconstruction strategies with synthetic data. The wavelength of the illumination is set to λ = 515 nm. Therefore, the diffraction limitation is 257.5 nm with NA = 1 and the maximum frequency of the pattern that can be projected on the object is $f_{obj}$ = $f_{DL}$ = 3.88 lp/μm. Then, $f_{img}$ = 0.1 lp/μm with $M_{2}$ = 40× and the period of the patten on the detector is $d_{img}=10\;\mu m=4d_{px}$ with $d_{px}$ = 2.5 μm, satisfying the discussed limitations during the illumination modulation process. Two illumination pulses’ structured patterns have modulation frequencies of $f_{obj}$ = 3.88 lp/μm and modulation angles of $-20^{\circ }$ and $70^{\circ }$. The above settings for simulations are the available upper bounds to explore the theoretical limitations of spatial resolution. For a practical system, the working distances of the projection and imaging optics to the induced phenomena in a sample are physically limited; therefore, NA has to be reduced to compromise with proper working distances.

The ground truth object’s frames carried by the illumination pulses are the upper and lower parts of a star resolution target with 50 spokes, as shown in Figure 3a. Figure 3b shows the overlapped FRAME image in the spatial and Fourier domains. The two frames are at distinct locations in the Fourier domain and are partially overlapped because high-frequency information is included during object mapping. With the traditional algorithm, two frames are recovered with a frame extraction radius of $R_{trad}=f_{obj}/2$, as shown in Figure 3c. Figure 3e shows the relative imaging modulation *M* curves for Frames 1 (red) and 2 (blue), implying the spatial resolution. *M* is defined as

(9) $$M=M_{rcv}/M_{gt}$$

where $M_{rcv}$ and $M_{gt}$ represent the modulations of the recovered and ground truth frames at an individual radius of the star resolution target. The *x*-axis represents the widths of a line pair ($d_{sr}$) at different radii and the *y*-axis represents the *M* values in percentage. The spatial resolution criterion is assumed to be the corresponding $d_{sr}$ when M=10%. In Figure 3e, the spatial resolution is approximately 520 nm at M=10%.

With the proposed FCS algorithm, two frames are recovered with a frame extraction radius of $R_{FCS}=f_{obj}$, as shown in Figure 3d. The relative modulation curves for Frames 1 and 2 reveal that the spatial resolution is 260 nm at M=10%, as shown in Figure 3f. Furthermore, compared to the traditional method, there are fewer reconstruction artifacts because the crosstalk from the zero-component is effectively blocked.

The simulation results are consistent with the theoretical analysis of the spatial resolution limitation of FRAME, which is the diffraction limitation for an ideal dual-frame system using the proposed FCS reconstruction method. Therefore, FRAME has the potential to be applied in ultrafast microscopy to provide multiple frames in a single laser-induced dynamic event.

### 3.2. Experiments

**Figure 4:**
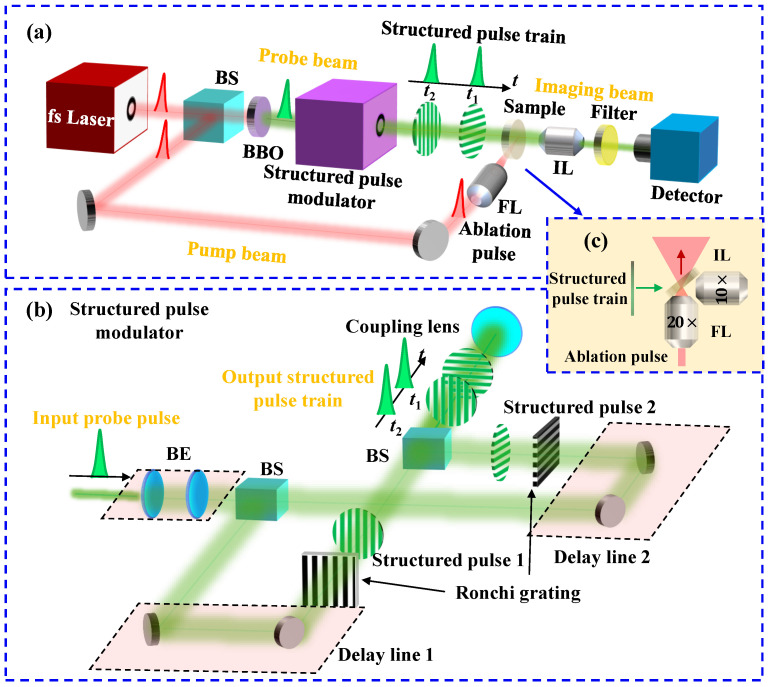
Diagrams of (**a**) the microscopic FRAME system for femtosecond laser ablation shadowgraphy, (**b**) the structured pulse modulator, and (**c**) the detailed sample configuration pointed by the blue arrow in (**a**). In (**c**), the green and red arrows represent the propagating directions of the structured pulse train and the ablation pulse, respectively. BS: beam splitter, FL: focusing lens, IL: imaging lens, BE: beam expander.

To experimentally demonstrate the spatial resolution of FRAME, a microscopic FRAME (micro-FRAME) system for femtosecond laser ablation shadowgraphy is constructed, as shown in Figure 4a, including a femtosecond laser source, an ablation pump beam, a structured probe beam, a fused silica glass sample, an imaging beam, and a detector. The femtosecond laser source (PH1-20, Light Conversion, Vilnius, Lithuania) generates pulses with a center wavelength of 1030 nm, a pulse width of 290 fs, and a pulse energy of 0.2 mJ.

An output pulse from the fs laser is directed into two beams by a beam splitter with T:R = 30:70 for the probe and pump beams, respectively. The ablation pulse in the pump beam undergoes precise temporal synchronization via a delay line before being focused onto the target through a focusing lens (FL, 20×, NA = 0.4) with a focal length of *f* = 10 mm to generate an ultrafast laser ablation phenomenon in the glass sample. To eliminate detection crosstalk from the pump laser, the probe pulse passes through a BBO crystal for second-harmonic generation (SHG), achieving high spectral isolation from the pump beam. The frequency-doubled probe pulse is then spatially encoded by a structured pulse modulator, generating a structured pulse sequence comprising two uniquely coded sub-pulses.

Figure 4b shows the details of the structured pulse modulator. The incident probe pulse is expanded with a beam expander to cover a proper illumination area and is equally divided into two sub-pulses with a beam splitter. Each sub-pulse passes through a precise delay line for temporal adjustment and is encoded spatially by a Ronchi grating ($f_{mask}$ = 0.04 lp/μm). The Ronchi gratings are rotated at $0^{\circ }$ and $90^{\circ }$ in the two channels. After the sub-pulses’ spatial modulation, they are recombined via a beam splitter to generate a pulse train with an adjustable pulse interval Δt, which is projected onto the target through coupling lenses, including a tube lens (*f* = 180 mm) and a microscope objective (20×, NA = 0.4, *f* = 10 mm). The projected patterns’ frequencies are $f_{obj}$ = 0.72 lp/μm with $M_{1}=0.056\times$.

Figure 4c shows the target configuration, where the glass plate is oriented at $45^{\circ }$ relative to the incident beam. The structured probe beam back-illuminates the ablation phenomenon induced by the ablation pump pulse. The probe and pump beams are orthogonal. The resulting probe patterns dynamically modulated by the ablation process are subsequently captured on a detector (2448 × 2048 pixels, $d_{px}=3.45$ μm) through a microscope objective (10×, NA = 0.25, *f* = 20 mm) with a diffraction limitation of 1.03 μm in the imaging pathway. The microscope objective ensures the high-contrast imaging of the patterns on the object with a frequency of $f_{obj}$ = 0.72 lp/μm, i.e., a period of 1.4 μm. A spectral bandpass filter with a center wavelength of 520 nm and a full width at half maximum (FWHM) of 20 nm is included in the imaging beam to block the noise from the pump laser and the induced plasma. The energy of the probe sub-pulse at the sample is approximately 5 μJ and the illumination area has a diameter of 130 μm. The energy of the pump pulse at the sample is approximately 80 μJ. The focus of this work is to explore the ultrafast imaging capabilities with high spatial resolution. The influence of the pump pulse’s energy on the sample will be studied in more detail in the future.

**Figure 5:**
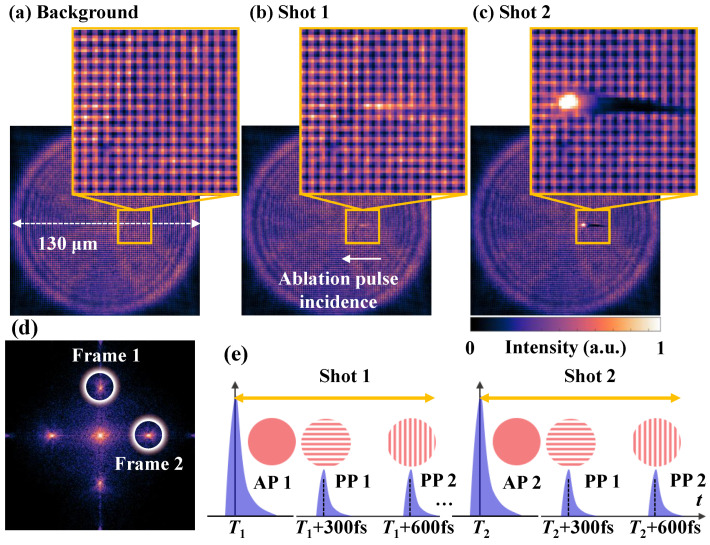
Micro-FRAME’s results for visualizing the glass ablation induced by the femtosecond pump laser. (**a**) Reference and Shot (**b**) 1 and (**c**) 2 ablation FRAME images in the spatial domain. (**d**) FRAME image (**a**) in the Fourier domain. (**e**) Temporal synchronization diagram of the measurements for Shots 1 and 2. In (**e**), the blue pulses and red patterns represent the temporal and spatial characteristics, respectively. AP: ablation pulse, PP: probe pulse.

The micro-FRAME’s results for visualizing the glass ablation induced by the femtosecond pump laser are shown in Figure 5. Figure 5a shows the reference FRAME image to calibrate the background when the pump laser is off. Figure 5b,c show the FRAME images carrying the dynamic evolution information when the pump laser interacts with the sample for Shots 1 and 2, respectively. The magnified views in Figure 5a–c reveal the ablation-induced phase modulations when the pump laser is off and on. The effective field of view (FoV) is determined by the overlapped region of the probe sub-pulses, which is 130 μm on the object plane. The illumination pulses’ modulation frequencies are $f_{obj}$ = 0.72 lp/μm and the orientation angles are $0^{\circ }$ and $90^{\circ }$ for the structured patterns encoded in the two channels. The arrow represents the incident direction of the ablation pump laser. The corresponding Fourier-domain representation of Figure 5a is shown in Figure 5d, revealing that the Fourier-domain information of the two channels resides in separable regions, labeled with white circles. From the previous theoretical analysis of spatial resolution, $f_{obj}$ = 0.72 lp/μm leads to a maximum spatial resolution of $d_{sr}$ = 1.4 μm.

The temporal synchronization relationship of the pump–probe measurements for Shots 1 and 2 is shown in Figure 5e. The two probe pulses sequentially illuminate the sample at 300 fs and 600 fs relative to the ablation pulse for Shot 1 at $T_{1}$ and Shot 2 at $T_{2}$. The two ablation shots interact with the sample at the same location with a time interval of 0.1 s.

**Figure 6:**
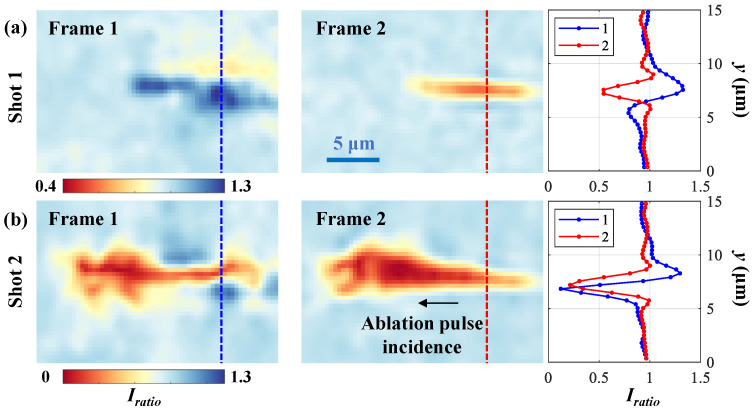
Recovered calibrated frames for Shots (**a**) 1 and (**b**) 2, including $I_{ratio}$ curves along the blue and red lines in Frames 1 and 2.

With the FCS reconstruction algorithm, the spatial-domain frames at 300 fs and 600 fs relative to the pump laser, Frames 1 and 2, denoted as ${\hat{I}}_{obj}^{n}$, can be extracted for each ablation shot. To calibrate the illumination background, ${\hat{I}}_{obj}^{n}$ is divided by the recovered frames ${\hat{I}}_{ref}^{n}$ from the reference frame image (Figure 5a). The calibrated frames can be expressed as $I_{ratio}^{n}={\hat{I}}_{obj}^{n}/{\hat{I}}_{ref}^{n}$, where *n* = 1 and 2. Figure 6a,b show the calibrated frames recovered from Figure 5b,c for Shots 1 and 2, respectively. The frame interval is 300 fs, corresponding to a frame rate of 3.3 Tfps. The colorbar represents $I_{ratio}$. When back-illumination is modulated by the induced dynamic scenes, $I_{ratio}<1$ and $I_{ratio}>1$ imply that the intensity of the probe pulse is dimmed and enhanced, respectively. $I_{ratio}$ curves along the blue and red lines in Frames 1 and 2 are plotted for each shot.

For Shot 1, there is an intensity-enhanced area ($I_{ratio}>1$) in Frame 1, which is caused by the sample’s refractive index change modulating the illumination’s distribution. In Frame 2, the probe pulse is partially absorbed by the induced plasma in a filament structure area (1.8 μm × 12 μm, height × width) with a minimum $I_{ratio}=0.5$.

For Shot 2, it is obvious that the penetration depth is deeper and the light absorption is stronger than those of Shot 1 along the ablation pulse’s propagation direction. In Frame 1, the narrow absorption area has a height of 1.4 μm. In Frame 2, the absorption is enhanced, leading to a minimum $I_{ratio}=0.1$.

## 4. Discussion

The experimental results demonstrate the capability of the micro-FRAME system in the applications of ultrafast laser processing, delivering a minimum spatial detail of 1.4 μm. The experimental setup is an implementation below the theoretical limitation demonstrated with simulations. The illumination pattern’s frequency on the object is $f_{obj}$ = 0.72 lp/μm, implying a maximum spatial resolution of 1.4 μm with the proposed FCS strategy, which is lower than the upper bound analyzed in the simulation $f_{obj}$ = 3.88 lp/μm, corresponding to a spatial resolution of 260 nm. To approach the theoretical limitation, the spatial resolution of micro-FRAME can be improved by generating patterns with a higher modulation frequency using high-frequency masks and high-NA coupling lenses in the structured pulse modulator. On the other hand, to increase the frame number in a single shot, more channels can be introduced in the illumination modulator. However, there is a compromise between the frame number and reconstruction quality. With the increase in the frame number, the frame distances in the Fourier domain will decrease, leading to more crosstalk from adjacent frames.

For structured illumination microscopy (SIM), structured illumination with multiple phases and orientations is used to achieve super-resolution imaging. The goal of SIM and FRAME algorithms is similar, which is unmixing the superimposed components in the Fourier domain. SIM is usually used in biological applications for relatively slow-varying scenes; therefore, it can capture multiple images sequentially with different illumination phases to separate the zero- and high-carrier-frequency information for the super-resolution reconstruction [36]. However, FRAME is a single-shot ultrafast imaging method and only one image is captured during the data acquisition with multiple illumination pulses. Extracting each frame’s superimposed information is an ill-posed inverse problem. The proposed FCS strategy can effectively remove the strong crosstalk from the zero-component. For the artifacts from the adjacent frames, iterative and deep learning algorithms developed in SIM [37] can be combined to ensure high-fidelity and high-spatial-resolution reconstruction with more frames, which will be investigated in the future.

For the temporal characteristics of FRAME, the frame exposure and frame interval depend on the sub-pulse width and adjacent sub-pulses’ interval, and they are insensitive to the spectral distributions. During the spatial modulation, the sub-pulses are slightly broadened after the propagation through the lenses. For instance, with BK7 lenses, the sub-pulse width can be broadened from 290 fs to ∼305 fs with a total propagation length of 100 mm at 515 nm. The stricter calibration of the temporal characteristics includes pulse width and interval measurements of the illumination sub-pulses output from the structured pulse modulator.

Furthermore, the micro-FRAME system implemented in this work is in a shadow imaging configuration. Reflective and phase imaging systems can be developed for visualizing laser-induced phenomena with various materials. The working assumption is consistent with the previous pump–probe methods used in laser–matter interaction visualization. The illumination sub-pulses’ power density should be much lower than the ablation threshold of the sample, inducing negligible transient phenomena compared to the pump pulse.

## 5. Conclusions

Single-shot ultrafast imaging techniques are desirable for visualizing non-repeatable dynamic events in diverse disciplines. Advanced approaches have emerged based on chirped or structured illumination for temporally resolved information encoding, which enables compressive acquisition and reconstruction of the 3D temporospatial signal. However, existing systems suffer from degraded spatial resolution, limiting applications in ultrafast laser processing, which demands micron and even sub-micron spatial resolution for visualizing the interaction areas on the scale of microns. To explore potential strategies for implementing single-shot femtosecond photography with high spatial resolution, the spatial resolution capability of the structured illumination methods (FRAME) was theoretically discussed. A frame extraction strategy (FCS) was proposed based on the Fourier conjugate symmetry, delivering an upper spatial resolution bound of the diffraction limitation. To verify the experimental feasibility, a two-channel microscopic FRAME system was constructed using high-frequency structured illumination (720 lp/mm) with a maximum spatial resolution of 1.4 μm. The system successfully captured the ablation process induced with a femtosecond laser in a glass plate with a frame interval of 300 fs and a field of view of 130 μm. The recorded frames reveal the distribution evolution of the induced plasma. This work provides a step forward for microscopic single-shot ultrafast imaging. By optimizing illumination with more channels and developing reconstruction algorithms using deep learning frameworks in the future, the spatial resolution and continuous recording capabilities of micro-FRAME can be improved, which is of great significance for further refined research on high-quality laser processing.

## Data Availability

The original contributions presented in this study are included in the article. Further inquiries can be directed to the corresponding author.
